# Applications of Data Mining Methods in the Integrative Medical Studies of Coronary Heart Disease: Progress and Prospect

**DOI:** 10.1155/2014/791841

**Published:** 2014-12-03

**Authors:** Yan Feng, Yixin Wang, Fang Guo, Hao Xu

**Affiliations:** ^1^Department of General Practice, Anzhen Hospital, Capital Medical University, Beijing 100029, China; ^2^Department of Cardiology, Xiyuan Hospital, China Academy of Chinese Medical Sciences, Beijing 100091, China

## Abstract

A large amount of studies show that real-world study has strong external validity than the traditional randomized controlled trials and can evaluate the effect of interventions in a real clinical setting, which open up a new path for researches of integrative medicine in coronary heart disease. However, clinical data of integrative medicine in coronary heart disease are large in amount and complex in data types, making exploring the appropriate methodology a hot topic. Data mining techniques are to analyze and dig out useful information and knowledge from the mass data to guide people's practices. The present review provides insights for the main features of data mining and their applications of integrative medical studies in coronary heart disease, aiming to analyze the progress and prospect in this field.

## 1. Introduction

Coronary heart disease (CHD) is a serious threat to human health, especially for the elderly. Integrative medicine (IM) specialists have accumulated a large number of data in the clinical practice of CHD, which contain important information about diseases, syndromes, syndrome diagnosis and thinking skills, prescription medication, treatment, prognosis and evolution syndrome, and other aspects of development trends. How to do our clinical researches relying on these objective, dynamically updated massive clinical data of IM for CHD, is the primary challenge for us [1–4].

At present, on current clinical research methods, due to the strict limitations in the included crowd and medication conditions for randomized controlled trials (RCTs), the studies have high internal validity but poor in external making difficulty for the findings in promoting practical application. On basis of practical international RCTs, real-world study (RWS) concepts and methods gradually rise, which is to reflect the real world as a whole through the “real world sample.” It is to choose interventions according to the actual condition and willingness of the patient and evaluate the effects of interventions with more comprehensive coverage of the crowd using statistical methods such as propensity score to control confounding bias. Thus, RWS has strong external validity than the traditional RCTs and can evaluate the effect of interventions in a real clinical setting. Therefore, the results are much closer to clinical practice. Integrative interventions emphasize individualized treatment, focusing on holistic, complex, and multiple effects in the evaluation of clinical efficacy. RWS undoubtedly opens up a new path for researches of integrative medical in CHD.

However, clinical data of IM in CHD are large in amount and complex in data types. All are multivalued and multitypes data, the attribute and label of each record have one or more options, and the clinical research data also have more confounding factors, making exploring the appropriate methodology become a hot topic.

Data mining is an interdisciplinary research field, which combines the latest research achievements such as statistics, data warehousing, information retrieval, machine learning, artificial intelligence, high performance computing, and data visualization. Data mining techniques are to analyze and dig out data useful information and knowledge from the mass data to guide people's practices, which is changing the use patterns of data with a new concept. Making the data mining techniques becomes a new researching way of RWS of IM for CHD.

To analyze clinical syndrome diagnosis and prescription experience of CHD in the real world with data mining methods, it cannot only find clinical rules and improve clinical diagnostic accuracy of CHD for IM physicians, but also get a deep understanding of IM academic thinking and grasp disease treatment rule. Therefore, using data mining methods for IM study in CHD will greatly improve the level of clinical diagnosis and treatment of IM study in CHD [5, 6] and has broad application prospects.

The main features of data mining include correlation analysis, classification and prediction, cluster analysis, and evolution analysis. In the field of exploring these features, RWS of IM in CHD has made considerable progress.

## 2. Correlation Analysis

Correlation analysis is to find interesting links between the association forms as X-Y items from large data, which can be interpreted as the probability that if X occurs, then Y also appears. Correlation analysis methods commonly include methods as association rule and complex network analysis. Data mining correlation analysis is widely used in etiology, clinical diagnosis, drug compatibility, and so on for IM clinical researches.

### 2.1. Association Rule

#### 2.1.1. Principle

It is a description for relationship between one thing and other associated or interdepended things [7–11], focusing on characterization [12] and the degree of association between the objects in the database [13]. Among the association rules mining, Apriori algorithm is the most basic, famous, and influential one. The core idea of the Apriori algorithm is a recursive method based on the frequency set theory, whose purpose is to dig out association rules with support and confidence no less than the minimum support threshold (min_sup) and minimum confidence threshold (min_conf) from the database.

#### 2.1.2. Characteristics

Interesting links hidden in large data can be showed by association rules or frequent item sets.

#### 2.1.3. Application Examples in CHD

A study found the most common syndromes of CHD after statistical analysis, which are mostly phlegm and blood stasis, water and blood stasis, and obstruction of coronary circulation syndromes; secondly common syndromes were syndrome of deficiency complicated with excessiveness, such as qi deficiency and blood stasis and yin deficiency with yang hyperactivity syndromes. The results reflect the thought of “two bu,” “three tong” in the treatment of CHD [14].

The association rule mining method was applied to a small sample study of CHD clinical data, using contingency table of definite probability method as a measure of association rules to find relationships between variables in small samples and get all two rules [15]. After a number of rules pruning based on the confidence, the results can more fully reveal the implications of the data information than that obtained by logistic regression method [16]. In the analysis of the law of prescription for famous TCM doctors with association rule, some basic recipes such as Huoxuetongmai agents, Shengmaisan, for treatment of CHD were also found [17]. There is theoretical and practical significance in how quickly and effectively mining association rules for some rational use from a massive database [18, 19].

### 2.2. Complex Network

#### 2.2.1. Principle

For the multiple elements of complex systems, it can be interconnected and form nodes due to certain inherent potential relationships. Most of the nodes have only a few connections, but some nodes have a lot of connections with other nodes. Complex system is a network system hosted by a few hubs, having a large number of functional groups composed with connecting hubs, which may reflect some or all of its overall characteristics [20–23]. Complex network is a method of data mining to dig out implicit, previously unknown, and potentially valuable to the decision-making relationships, patterns, and trends from large data [24].

#### 2.2.2. Characteristics

This method can find potential links between two or more elements of complex systems, showing the relationship between picture elements.

#### 2.2.3. Application Examples in CHD

Using cross-sectional survey, the study collected the clinical information of 3018 hospitalized CHD patients through individualized information acquisition platform of CHD. The relationships among syndrome, therapeutic treatment, and Chinese herbs were excavated by means of complex networks based on theory of correspondence between prescription and syndrome.

It found that the fundamental syndrome factors were blood stasis, qi deficiency, phlegm-turbid, yin deficiency, yang deficiency, qi stagnation, and blood deficiency. The therapeutic treatment mainly included activating blood circulation, clearing heat, invigorating qi, resolving turbid and phlegm, nourishing yin, warming yang qi, and dispersing obstruction. These methods constituted an association with major syndrome factors. The major syndrome factors constituted an association with the following Chinese herbal medicines: Huang qi (Radix Astragali Mongolici), Chen pi (Pericarpium Citri Reticulatae), Di Huang (Radix Rehmanniae), Chuanxiong (Rhizoma Chuanxiong), Baizhu (Rhizoma Atractylodis Macrocephalae), Taoren (Semen Persicae), Fuling (Poria), Gan cao (Radix Glycyrrhizae), Ban Xia (Pinellia Ternate), Ze Xie (Rhizoma Alismatis), Chi Shao (Radix Paeoniae Rubra), Dang Gui (Radix Angelicae Sinensis), Danshen (Radix Salviae Miltiorrhizae), Zhi Qiao (Fructus Aurantii Submaturus), Gui Zhi (Ramulus Cinnamomi), and Mai Dong (Radix Ophiopogonis Japonici). The efficacies of Chinese herbal medicines associated with syndrome factors mainly include alleviating pain, resolving turbid and phlegm, clearing heat, activating blood circulation, invigorating qi, cooling blood, promoting urination, resolving stagnation, removing toxic material, nourishing blood, regulating qi, quieting spirit, invigorating spleen, regulating menstruation, promoting defecation, moistening dryness, and resolving stasis.

The therapeutic methods for CHD are based on consistency in theory, method, formula, and medicines. The application of therapeutic methods for clearing heat and removing toxical material was compared relatively more with other methods, so it is necessary to separate heat as the complement blood stasis, phlegm, and qi stagnation syndromes to more fully reflect evolution and characteristics of CHD syndromes [25].

## 3. Classification and Prediction

Classification and prediction are two forms of data analysis. Classification is to analyze training data set to identify the typical characteristics of data in the same class based on the characteristics of data and use them to classify new data. The key of classification is to export functions or models for classification. There is a long time to study the issue and put forward many methods used to derive the classification models, including decision tree classification and neural network. Classification is for predicting discrete categories of data objects; when the prediction data objects are not a class but continuous value, it is often called prediction.

The data mining functions of classification and prediction is widely used in medical diagnostics, disease risk prediction and other fields for clinical practice of IM.

### 3.1. Decision Tree

#### 3.1.1. Principle

It is a data classification process through a series of rules [26]. Determined by a series of “if then” logic (branching) relationships, this method inference a set of classification rules from a set of no order and no rules examples, and express the distribution probabilities of all possible outcomes 9 with a tree chart as the decision tree, so as to achieve the purpose of predicting accurately or correct classification [27].

Decision tree is being used more and more in clinical studies, especially in the clinical diagnosis [28]. In IM researches, the tree model is mainly applied in standardization of syndromes characteristics and diagnosis [29], setting up medical model [30], influencing factors of syndrome changes, and evaluation on the efficacy of IM researches [31].

#### 3.1.2. Characteristics

This method combines the disorder existing data together and build relationships connected layer upon layer to classify and predict the targets or outcomes.

#### 3.1.3. Application Examples in CHD

Decision tree pattern could identify phlegm-blood stasis syndrome of unstable angina patients clearly and more intuitively, and it also could self-extract recognition rules. It had advantage in the data mining of syndrome-clinical physicochemical index corresponding pattern [32–35]. Besides that, tree structure models were built to summarize the correspondence between qi deficiency syndrome and physicochemical index based on *t* test, nonparametric analysis, and Spearman correlation analysis. The study found that the accuracy identification rate of tree structure model of qi deficiency syndrome with six core indexes, such as EF and P-R interval, was 77.78%. Decision tree model can identify qi deficiency syndrome of CHD patients with diabetes clearly and more intuitively. Decision tree model is a promising method in data mining of qi deficiency syndrome and index association patterns [36].

### 3.2. Artificial Neural Network

#### 3.2.1. Principle

Artificial neural network, also known as connection machine model, is produced on interdisciplinary researches in modern neurology, biology, psychology, and so forth. As a computing system developed on the basis of simulation of human brain tissue, it reflects the fundamental process of processing external things by biological neural system. It is a network system composed of a large number of interconnected processing with basic characteristics of the biological nervous system. It is reflects the several brain functions to a certain extent, a kind of simulation to biological system, having the abilities of nonlinear mapping and learning, adaptability, fault tolerance, and associative storage.

#### 3.2.2. Characteristics

The independent variables may be continuous in the model application or may be discrete, regardless of whether the normality of variables and independence between variables and other conditions are satisfied. It can identify the complex nonlinear relationships between the variables; especially when using conventional methods cannot achieve the purpose of statistical analysis or ineffective, this model can often receive good results.

#### 3.2.3. Application Examples in CHD

Based on the clinical epidemiology investigation in coronary heart disease, the study constructed an artificial neural network model of the CHD of Chinese medicine syndromes on the basis of neural network toolbox, and then it tests the performance of this model using a retrospective of inspection and prospective testing method. The diagnostic accuracy of rate is 90.5% in 496 cases of already collected of retrospective examination showed, and specific syndrome types of discrimination and sample number accuracy were positively correlated. The diagnostic accuracy of rate is 91.36% in new collection of 132 cases of the prospective examination showed. It is thought that the artificial neural network can better the forensics of syndrome because it can explore the internal rules of TCM syndrome. And there is a good prospect using artificial neural network in the study of standardization of traditional Chinese medical syndrome [37].

## 4. Cluster Analysis

Cluster analysis is to divide physical or abstract objects into several groups or classes based on “greatest intragroup similarity, smallest intergroup similarity” principle. Clustering is unsupervised learning, and the input set is a set of nonpredefined classes records without any classification. Good clustering method ensures that intragroup similarity is very high and intergroup similarity is very low. Cluster analysis function of data mining is widely used in data integration, analysis of clinical features, and other aspects in the field of IM for CHD.

### 4.1. Clustering Analysis

#### 4.1.1. Principle

It is a kind of mathematical statistics for the study of “like attracts like.” Cluster analysis can put some of the observed objects to be classified according to certain characteristics, and there is a wide range of applications in biology and medical classification problems [38].

#### 4.1.2. Characteristics

It can do some basic classification for hybrid data to make it well-organized and easy to find data characteristics.

#### 4.1.3. Application Examples in CHD

With cluster method to analyze the database, we summarize the syndromes features of four periods: (1) early onset period: qi open-minded syndrome and qi and yin deficiency syndrome, (2) paroxysm period: qi stagnation and phlegm obstruction syndrome, yang malaise heart syndrome, cold coagulation heart vessel syndrome, and blood stasis yang syndrome, (3) remission period: liver and spleen no coordination syndrome, yang deficiency of heart and kidney syndrome, and cardiopulmonary qi deficiency syndrome, and (4) recovery period: heart qi deficiency syndrome, yang deficiency and qi stagnation syndrome, and qi and yin deficiency syndrome. That enriches the content of CHD syndromes [39].

The K-center clustering method was adopted for the analysis of clinical data and syndrome information of 154 cases of prethrombosis state. The results showed that traditional clinical syndrome differentiation presented 12 syndrome patterns, and they were blood stasis syndrome, qi deficiency syndrome, damp turbidity syndrome, yin deficiency syndrome, yang deficiency syndrome, phlegm turbidity syndrome, damp-heat (toxicity) syndrome, qi stagnation syndrome, blood deficiency syndrome, phlegm heat syndrome, and cold accumulation syndrome. Among the 12 patterns, the blood stasis syndrome and qi deficiency syndrome were more commonly seen than other syndromes, accounting for 49.1%, and cold accumulation syndrome was most rarely seen. Syndrome clustering analysis results presented 4 syndrome patterns, yang deficiency and blood stasis accounted for 60.4%, syndrome of phlegm damp aggregated with heat and qi stagnation accounted for 20.1%, qi and yin deficiency syndrome accounted for 13.0%, and cold accumulation syndrome accounted for 6.5%. Yang deficiency and blood stasis syndrome was the most common type. Cluster analysis is thought to be helpful for the research of Chinese medical syndrome and can provide reliable basis for syndrome differentiation, which will lay the foundation for IM treatment and efficacy evaluation [40].

### 4.2. Shannon Entropy Mutual Information

#### 4.2.1. Principle

It is such a complex system divided manner with probability entropy as premise to extract feature combinations with maximum information by calculating a correlation coefficient for each variable and the other variables.

#### 4.2.2. Characteristics

This method is not entirely dependent on the frequency, but also on the two variables appearing or not simultaneously to characterize the correlation between the two aspects. This method is used not only to deal with linear data, but also to deal with nonlinear data.

#### 4.2.3. Application Examples in CHD

The data mining technology based on the Shannon entropy mutual information was used to analyze the complicated correlations of the statistical distribution of CHD syndromes associated physical and chemical indexes. It was found that 7 in 13 syndrome factors including qi deficiency, blood stasis, turbid phlegm, yin deficiency, cold coagulation, yang deficiency, and qi stagnation which have been most researched were involved in about 134 physical and chemical indexes mentioned in the literature. It obtained the ranked top 10 physical and chemical indexes of each syndrome factors, after analysis calculation. The study suggested there were perplexing relations with Chinese medical syndromes and physical and chemical indexes, which can be revealed by the data mining technology based on the Shannon entropy mutual information [41].

## 5. Evolutionary Analysis

Evolutionary analysis is to describe and model following the changing laws or trends of time-varying objects, including data analysis as time series, sequence, or cycle matching pattern. The evolution of data mining analysis capabilities in the field of IM is widely used to predict clinical outcomes and evaluate the efficacy of clinical programs.

Technologies used in the studies of CHD by IM include Bayesian network, support vector machine, Markov model, and random walk model.

### 5.1. Bayesian Network

#### 5.1.1. Principle

Based on Bayes' theory,* Bayesian Network* has solid foundation of statistical theory and strong integration capabilities for the sample information and prior knowledge. As one of the important data mining algorithms for classification [42],* Bayesian Network* is an ideal reasoning method of uncertainty researches, which can give the probability for samples belonging to a specific class and also minimize rates of error and risk in the reasoning process.

If the likelihood of event results cannot be predicted, then* Bayesian Network* is the only way to quantify that the probability is to obtain the occurrence of the event. Bayesian classification is a typical statistical classification method. The common practice is to establish a link of the prior probability and posterior probability of the event and then to determine the category to the largest posterior probability sample through the judgment of posterior probability.

#### 5.1.2. Characteristics

The occurrence probability of final outcome is calculated by existing data.

#### 5.1.3. Application Examples in CHD

Based on Bayesian network, a paper studies the construction of Chinese medical clinical diagnosis model for CHD, and gain information algorithm is used to choose the fields used for the two models. The experimental data are selected from the electronic patient information database, and the experimental results show that Bayesian networks have better classification capability in Chinese medical clinical diagnosis mode [43].

With syndrome factors and combination law as the observing points, another study used Bayesian network to do some qualitative and quantitative researches for syndrome factors and dig CHD syndrome from the prominent Chinese medical doctors database and achieved good results. Besides the above, it also used in blood stasis syndrome differentiation and setting up CHD diagnosis model [44–46].

### 5.2. Support Vector Machine (SVM)

#### 5.2.1. Principle

As a monitoring statistical learning method based on the structural risk minimization principle,* SVM* can get a globally optimal solution without the need to seek prior probability. It can use a certain preselected nonlinear mapping to make the input vectors mapped to a high dimensional feature space and in the high dimensional feature space to construct the optimal separating hyperplane, finally, to do classification making use of the hyperplane.

#### 5.2.2. Characteristics

It is a statistical learning model under recognized high-dimensional and small sample sizes and is generally applicable in classification and regression studies. Its trained models have the characteristics of global optimum, and as long as the parameters {*C*, *σ*} are the same, the results will remain stable and consistent training [47, 48].

#### 5.2.3. Application Examples in CHD

A study set up a database of diagnosing and treating CHD and was on the basis of 115 typical medical records from prominent Chinese medical doctors. The syndrome factors and relevant studies were analyzed and conducted by using SVM. It found that there were mainly 8 syndrome factors draw, including blood stasis, turbid phlegm, qi deficiency, yang insufficiency, yin deficiency, inner heat blood deficiency, and qi stagnation. The quantitative diagnosis was confirmed and CHD characteristics were explained. The laws of medicate administration for abovementioned 8 syndrome factors were summed up from prominent Chinese medical doctors for treating CHD [49].

### 5.3. Partially Observable Markov Decision Process (POMDP)

#### 5.3.1. Principle

POMDP model is a dynamic decision model based on Markov process promoted by the Russian mathematician Markov after some improvements, which is the most common method in a dynamic programming strategy. Its purpose is to seek the best solution in many prescriptions applying optimization techniques [50].

#### 5.3.2. Characteristics

Data acquisition is performed in parallel with the operation process, and the entire data acquisition does not require large-scale acquisition process as long as the regulation of data entry has been established, which greatly saves the cost of the study to facilitate the continuous optimization of the prescriptions. Furthermore, this dynamic process is a combination of man and machine model, and the strict mathematical operation is carried out at the same time while doing empirical evaluation.

#### 5.3.3. Application Examples in CHD

Based on existing data, applying POMDP to compare the prescriptions of patients with same TCM syndrome element and no long-term end-point event, it was found that optimizing prescription recommendation for “qi deficiency” patients is “Milkvetch root + Si junzi decoction without Radix Glycyrrhizae,” prescription of “blood stasis” recommended: “Danshen root + Taohong Siwu decoction plus orange fruit without rehmanniae radix,” and prescription of “turbid phlegm” recommended: “Gualou xiebai banxia decoction plus dried tangerine peel, Largehead Atractylodes rhizome, Platycodon root.” The prescriptions derived from real clinical data are experiences and summaries of clinical practice with considerable clinical significance. The proposals using this rigorous mathematical comparison method are in conformity with the clinical normal circumstances and prove the reliability and operability of optimizing prescription method in efficacy evaluation on the other hand [51].

### 5.4. Random Walk Model

#### 5.4.1. Principle

*Random Walk Model* is a commonly mathematical model simulating the statistical mathematics to provide the best possible state. Random walk model is a way of exploring the movement of things that set probability theory and dissipative structure theory in one. Its basic idea is, given a particle in space, and its moving vectors in space (including the direction and distance) are controlled by a random amount of transition probabilities, which can simulate complex process, such as the molecular Brownian motion of nature and electronic random motions in the metal.

#### 5.4.2. Characteristics

It can compare and evaluate treatment options based on the dynamic changes after the intervention.

#### 5.4.3. Application Examples in CHD

The study evaluates the clinical effects of Shengmai injection in treating CHD based on correct syndrome differentiation and incorrect syndrome differentiation and found that there were 273 patients in the correct syndrome group and 4 patients died (case-fatality rate was 1.47%). There were 297 patients in the incorrect syndrome group and 7 patients died (case-fatality rate was 2.36%). In the correct syndrome group, random fluctuation peak of comprehensive evaluation index, walk steps, positive growth rate of walk, ratio, random fluctuation power law, increase rate, and record times of comprehensive evaluation index were 1472, 13617, 0.1081, 9.25, 0.6742, 0.4706, and 3128, respectively, while, in the incorrect syndrome group, 1030, 14588, 0.0706, 14.16, 0.6606, 0.3128, and 3293, respectively. The random fluctuation power law in both groups exceeded 0.5. There is a long-range correlation between the comprehensive evaluation index and therapeutic method in the CHD patients were treated with Shengmai injection. The clinical therapeutic effects of Shengmai injection under correct syndrome differentiation are better than the effects of Shengmai injection under incorrect syndrome differentiation [52].

## 6. Other Functions: Text Mining

It is a direction of data mining, in which you can find potential patterns and trends from millions of text data. In the field of IM, text mining can discover knowledge from amounts of literatures in order to promote development of clinical research and treatment programs in IM and provide new ideas and ways for IM studies with more objective and repeatable results [53].

### 6.1. Principle

text mining method is to find and mine inductive knowledge from the texts such as useful models, trends, and rules [54–57]. The text knowledge discovery technology, which is text mining technology, is the product of artificial intelligence, machine learning, natural language processing, data mining, and related automatic text processing such as information extraction, information retrieval, and text classification. Information extraction positions target data units from natural language texts and put the unstructured free texts into structured data that meet the application of requirements, which is extract free text data to fill predefined structured templates.

Traditional machine learning methods described above, such as neural networks, Bayes network, decision tree, k-nearest neighbor, and support vector machine, are all used for text classification and archiving [58]. The recent application of a relatively new model in diagnosis and treatment in the field of IM for CHD is the topic model technology.

### 6.2. Topic Model

#### 6.2.1. Principle

As the product of text mining and natural language processing technology in recent years,* Topic Model* is a statistical model that can extract a class of topics implicit in the documentation set (not limited to text documents, it can be other discrete data sets), each of which is distribution of some words with related semantics. Topic models use the topic ideas of the text to make the text from the high dimension of the word to the low dimension of the topic, which reduces the high-dimensional sparse feature of the text and the effect of noise word processing in text information, as well as capturing the semantics of the text.

#### 6.2.2. Characteristics

The characteristics of topic model are (1) easy for effective representation, organization, and storage of the text; (2) easy for semantic information retrieval, information extraction, automatic summarization extracts, and other operations according to the semantics; and (3) easy for effective text classification and clustering.

#### 6.2.3. Application Examples in CHD

Based on topic model, the study analyzed the accompanied syndromes, comorbidities, and usage of Chinese herbal for preliminary optimization of the treatment regimen in different of accompanied syndromes and complication.

Seeing from the experiments of topic model, the obtained results are consistent with the actual situation in which the model can get the hierarchical relationship of the data. To set the accompanied syndromes and complications of a patient, the model can predict the corresponding use of Chinese herbs. Similarly, to give a combination of some Chinese herbs, the model can infer what accompanied syndromes and complication of the patient are. Topic model can extract the regularity of treatment regimens with clinical significance, provide a novel theoretical approach for the study of treatment regimen optimization, give objective evidence for the clinical syndrome and disease differentiation treatment, and set a new statistical analysis method for analysis of prescription with varied syndromes and diseases [59].

## 7. Conclusions

Real-world studies of modern medicine in CHD have got a rapid development in recent years with a wide range and large-scale covering and formed a multicountry research participation model such as GRACE. In contrast, real-world study of CHD in IM is just at the beginning, and the studies based on more than a million cases of CHD have not been reported, particularly lacking suitable real-world research methodology system of IM. It is undoubtedly a useful exploration to introduce cutting-edge technologies—data warehousing and data mining in the field of information to IM clinical studies, and establish massive data based, data-driven clinical research model to solve technical bottlenecks in IM clinical researches with individual clinics as a feature.

However, the application of data mining methods in clinical intervention to CHD for RWS problems of IM has established a relatively complete paradigm and technical support currently but is still in the developing stage, and its own methodology and practical application are continuously improved. How to get mining methods and clinical practice closely combined, how to better interpret and apply data mining results, and how to better improve the traditional mining methods also need further exploration to seek better solutions. Following the general rules of data mining methods [60], combining it with the characteristics of clinical practice in IM [61–63], continuing to explore suitable data mining methodology, and getting continuous optimization improvement in practice on basis of the existing database are the development direction of RWS of IM for CHD in the future.
